# Urgent Resection of Hepatocellular Carcinoma With Intracardiac Extension Under Moderate Hypothermic Circulatory Arrest

**DOI:** 10.1093/icvts/ivag192

**Published:** 2026-07-01

**Authors:** Masaaki Kobayashi, Satoshi Kuroyanagi, Onichi Furuya, Akinao Miki

**Affiliations:** Department of Cardiovascular Surgery, Kishiwada Tokushukai Hospital, Osaka 596-0042, Japan; Department of Cardiovascular Surgery, Kishiwada Tokushukai Hospital, Osaka 596-0042, Japan; Department of Cardiovascular Surgery, Kishiwada Tokushukai Hospital, Osaka 596-0042, Japan; Department of Cardiovascular Surgery, Kishiwada Tokushukai Hospital, Osaka 596-0042, Japan

**Keywords:** hepatocellular carcinoma, intracardiac extension, inferior vena cava, right atrium, moderate hypothermic circulatory arrest

## Abstract

Intracardiac extension of hepatocellular carcinoma (HCC) via the inferior vena cava (IVC) is uncommon and may result in fatal cardiac complications. We report a case of urgent cardiac surgery performed to prevent imminent cardiac deterioration and facilitate subsequent multidisciplinary treatment. A 64-year-old man with a history of right hepatectomy for stage III HCC and radiofrequency ablation for recurrent disease was admitted following a traffic accident. Echocardiography revealed a 4.6 × 3.6 cm right atrial mass continuous with the IVC. Although the patient had no overt cardiac symptoms, frequent episodes of non-sustained ventricular tachycardia and bilateral lower extremity oedema raised concern regarding impending cardiac complications. Urgent tumour resection was performed under moderate hypothermic circulatory arrest (lowest rectal temperature, 32 °C). Following cardiac and orthopaedic surgery, the patient underwent transcatheter arterial chemoembolization and systemic therapy with durvalumab plus tremelimumab. At a 1-year follow-up, no intracardiac recurrence was observed, pulmonary metastatic lesions had regressed, and the patient remained clinically stable. Urgent cardiac surgery may serve as a bridge to multidisciplinary treatment in selected patients with HCC and intracardiac extension.

## INTRODUCTION

Hepatocellular carcinoma (HCC) with extension into the inferior vena cava (IVC) and right atrium is uncommon and is associated with poor prognosis because of the risk of fatal cardiac complications.^1^^,^^2^ Although systemic therapy is currently the standard treatment for advanced HCC,^3–5^ intracardiac tumour extension may require urgent intervention when life-threatening cardiac events are anticipated. We report a case of HCC with intracardiac extension managed by urgent cardiac surgery followed by multidisciplinary treatment, resulting in favourable cardiac and oncologic outcomes at 1-year follow-up.

## CASE REPORT

A 64-year-old man underwent right hepatectomy for stage III HCC in 2013 and radiofrequency ablation for recurrence in 2014 but discontinued follow-up. In September 2024, he was admitted after a traffic accident with a left lower extremity fracture.

**Figure 1.**
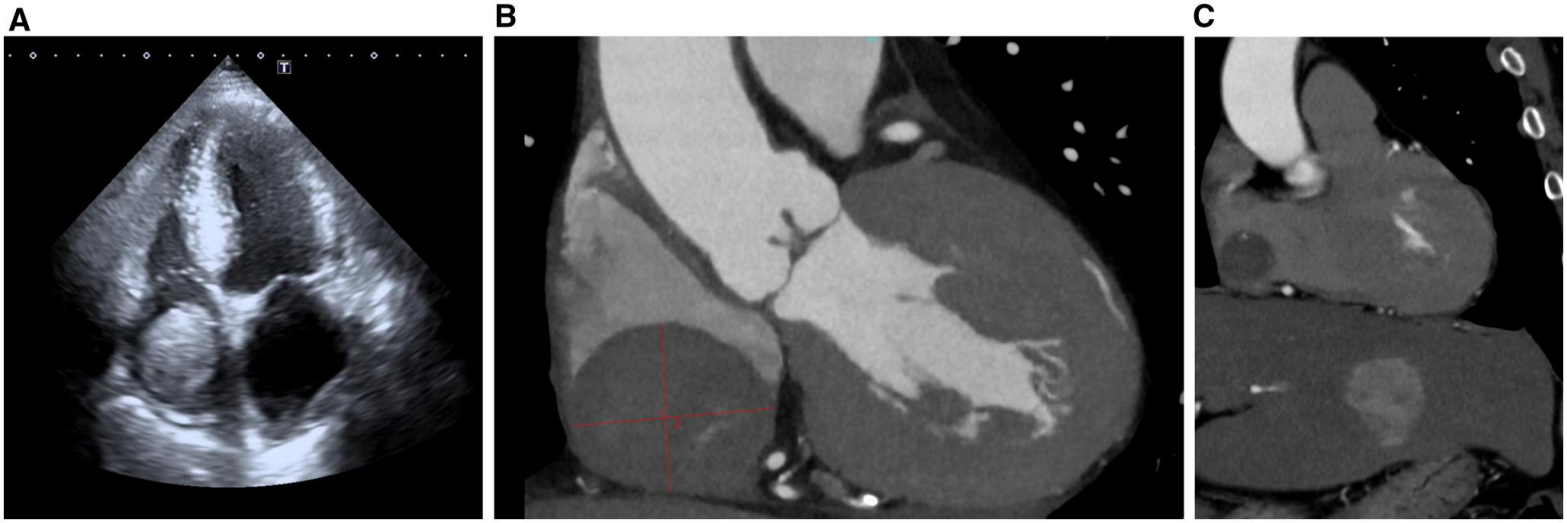
Preoperative Images. (A) Preoperative TTE, (B) Preoperative CT, (C); Liver CT.

Preoperative transthoracic echocardiography incidentally revealed a 4.6 × 3.6 cm mass (**Figure 1A**) occupying the right atrium. Contrast-enhanced computed tomography demonstrated continuity between the right atrial mass and the IVC (**Figure 1B**), consistent with intracardiac extension of HCC. Recurrent lesions were also identified in the remnant liver (**Figure 1C**). Liver function was preserved (Child–Pugh grade A).

Although the patient had no overt cardiac symptoms such as syncope or dyspnoea, frequent episodes of non-sustained ventricular tachycardia were documented during hospitalization. In addition, bilateral lower extremity oedema was present, suggesting impaired venous return related to IVC involvement. Given the large intracardiac mass and the risk of tricuspid obstruction or embolization, urgent surgical intervention was deemed appropriate.

Median sternotomy was performed. Cardiopulmonary bypass was established via ascending aortic and bicaval cannulation. Moderate hypothermic circulatory arrest was instituted to facilitate safe tumour resection, with a lowest rectal temperature of 32 °C. Cardiopulmonary bypass time was 109 min, aortic cross-clamp time was 70 min, and circulatory arrest time was 33 min. Total operative time was 3 hours and 27 min.

The right atrial mass and contiguous IVC tumour thrombus were resected en bloc, including partial IVC wall resection followed by reconstruction (Video 1). Histopathological examination confirmed HCC involving the right atrium through direct extension from the IVC.

**Figure 2.**
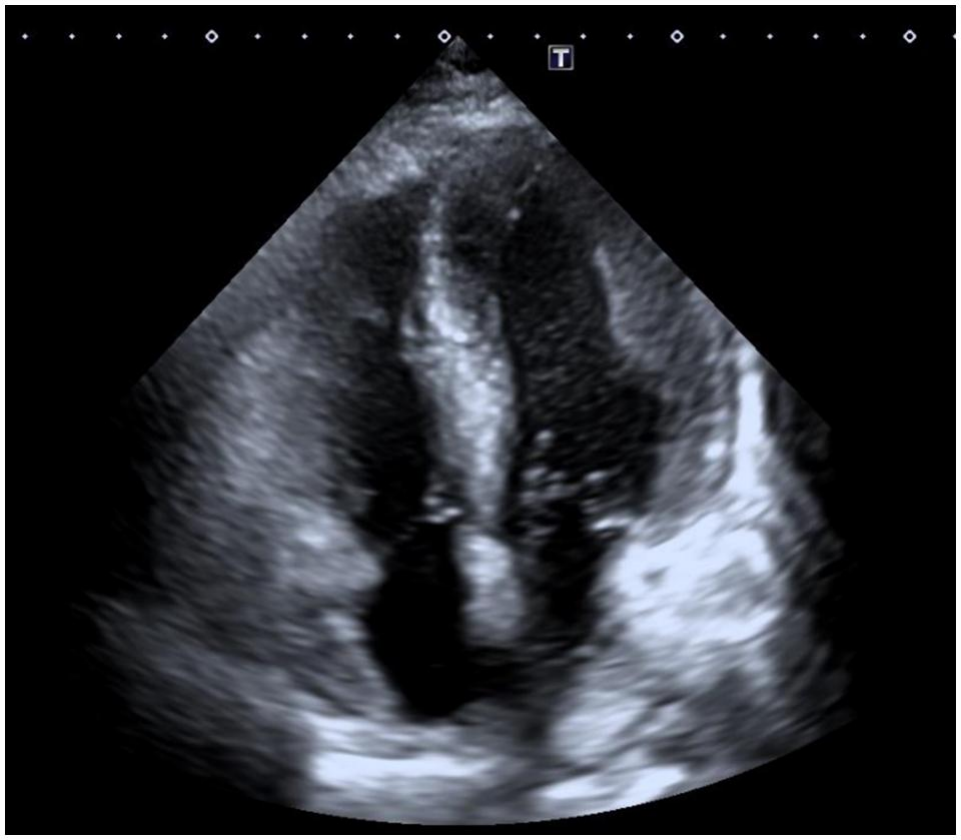
Postoperative TTE.

The postoperative course was uneventful, and no neurological complications occurred. Postoperative transthoracic echocardiography demonstrated no residual intracardiac mass (**Figure 2**).

Following recovery from cardiac surgery, the patient underwent orthopaedic surgery for the lower extremity fracture. Subsequently, transcatheter arterial chemoembolization was performed for recurrent hepatic lesions, followed by systemic treatment with durvalumab plus tremelimumab. At 1-year follow-up, no intracardiac recurrence had been identified. In addition, pulmonary metastatic lesions had regressed, and the patient remained clinically stable.

## DISCUSSION

Hepatocellular carcinoma with extension into the IVC and right atrium is uncommon and is associated with a poor prognosis because of both advanced tumour biology and the risk of fatal cardiac complications.^1^^,^^2^ Although systemic therapy remains the standard treatment for advanced HCC,^3^ intracardiac extension represents a unique clinical situation in which immediate management of the cardiac lesion may take priority over oncologic considerations.

In the present case, the indication for surgery was the prevention of potentially catastrophic cardiac events rather than oncologic cure. Although the patient did not exhibit overt haemodynamic instability, frequent episodes of non-sustained ventricular tachycardia and bilateral lower extremity oedema, suggesting impaired venous return, raised concern regarding imminent clinical deterioration. In addition, the large intracardiac mass extending continuously from the IVC posed a risk of tricuspid obstruction and embolization. These findings supported the decision to perform urgent surgical intervention.

Another important issue is the choice of operative strategy. Moderate hypothermic circulatory arrest facilitated controlled en bloc resection under direct visualization while minimizing the risk of tumour fragmentation and embolization. Although alternative approaches, including normothermic cardiopulmonary bypass with caval control, have been reported,^1^^,^^2^ the extent of intracardiac involvement in this patient favoured the use of circulatory arrest. No neurological complications occurred postoperatively.

The principal message of this case is not that surgical resection is technically feasible in patients with HCC extending into the right atrium, as this has already been described.^1^^,^^2^ Rather, this case demonstrates that urgent cardiac surgery may serve as a bridge to contemporary multidisciplinary treatment. Following cardiac surgery, the patient successfully underwent orthopaedic treatment, TACE for recurrent hepatic lesions, and systemic immunotherapy with durvalumab plus tremelimumab. At 1-year follow-up, no intracardiac recurrence was observed, pulmonary metastatic lesions had regressed, and the patient remained clinically stable. Immune checkpoint inhibitor-based therapies, including atezolizumab plus bevacizumab and durvalumab plus tremelimumab, have improved outcomes in advanced HCC.^4^^,^^5^ In selected patients presenting with imminent cardiac complications, surgery may therefore provide an opportunity to access effective subsequent therapies rather than functioning solely as a palliative intervention.

## Data Availability

All relevant data are within the manuscript and its Supporting Information files.

## References

[1] Wang Y, Yuan L, Ge RL, Sun Y, Wei G. Survival benefit of surgical treatment for hepatocellular carcinoma with tumor thrombus in the inferior vena cava or right atrium. Ann Surg Oncol. 2013;20:3709-3717. 22956071 10.1245/s10434-012-2646-2

[2] Pesi B, Ferrero A, Grazi GL, et al Surgical management of hepatocellular carcinoma with tumour thrombus in the inferior vena cava and right atrium. Ann Surg Oncol. 2015;22:394-401.

[3] European Association for the Study of the Liver. EASL Clinical Practice Guidelines on the management of hepatocellular carcinoma. J Hepatol. 2025;82:315-374. 39690085 10.1016/j.jhep.2024.08.028

[4] Finn RS, Qin S, Ikeda M, et al; IMbrave150 Investigators. Atezolizumab plus bevacizumab in unresectable hepatocellular carcinoma. N Engl J Med. 2020;382:1894-1905. 32402160 10.1056/NEJMoa1915745

[5] Abou-Alfa Ghassan K, Lau George, Kudo Masatoshi, et al Tremelimumab plus durvalumab in unresectable hepatocellular carcinoma. NEJM Evid. 2022;1:EVIDoa2100070. 38319892 10.1056/EVIDoa2100070

